# Sufficient Reasons to Act Wrongly: Making Parfit’s Kantian Contractualist Formula Consistent with Reasons

**DOI:** 10.1007/s11406-016-9766-z

**Published:** 2016-09-23

**Authors:** Mattias Gunnemyr

**Affiliations:** Department of Philosophy, The Faculties of Humanities and Theology, Lund University, Box 192, Helgonavägen 3, 221 00 Lund, Sweden

**Keywords:** Parfit D, *On What Matters*, Kantian Contractualist Formula, The Consent Principle, Reasons

## Abstract

In *On What Matters* (2011) Derek Parfit advocates the Kantian Contractualist Formula as one of three supreme moral principles. In important cases, this formula entails that it is wrong for an agent to act in a way that would be partially best. In contrast, Parfit’s wide value-based objective view of reasons entails that the agent often have sufficient reasons to perform such acts. It seems then that agents might have sufficient reasons to act wrongly. In this paper I will argue that such reasons are a symptom of a fundamental inconsistency between the Kantian Contractualist Formula and Parfit’s view of reasons. The formula requires that we consider what *everyone* could rationally will, while a wide value-based objective view requires that we consider only what *the agent* has sufficient reasons for doing. The same inconsistency is particularly obvious in Parfit’s version of the Consent Principle, which share important features with the Kantian Contractualist Formula. Parfit accepts that moral principles might entail that we sometimes have sufficient reasons to act wrongly. However, to accept that supreme moral principles have such implications is objectionable if you, like Parfit, also hold that principles with such implications should be rejected or revised. I suggest that we could abandon the requirement that we have to consider the reasons of everyone. This would make the Kantian Contractualist Formula consistent with Parfit’s view of reasons, at least in this respect. I also argue that we can keep most implications of the Kantian Contractualist Formula that Parfit finds attractive.

## Introduction

Derek Parfit’s influential work *On What Matters* has been widely discussed, but so far the commentary has largely revolved around the metaethical views it presents. I want instead to address some concerns about the central moral principles advocated in this work. As I see it, these principles can be shown to be inconsistent with certain implications of his metaethical view in some important cases. Parfit advocates three central moral principles, all of which are revised versions of Kantian Contractualism, Scanlonian Contractualism and Rule Consequentialism. He argues that the three principles ultimately amount to one and the same claim. He then unites them in what he calls the Triple Theory. This paper only treats Kantian Contractualism and the related Consent Principle, but I think the worries I raise here trouble the other formulas as well.

Parfit rejects or revises moral principles in order to make them less conflicting with what we have reasons to do. Most significantly, he revises Kant’s own moral formulas to make them less vulnerable to some important objections. He ends up with the *Kantian Contractualist Formula* that “Everyone ought to follow the principles whose universal acceptance everyone could rationally will” (Parfit 2011 – henceforth referred to by page number alone – pp. 20, 342, 355, 378). Whether this formula is truly Kantian can be disputed, but Parfit argues that our primary aim as philosophers should be to make progress – not merely to arrive at correct interpretations.

The Kantian Contractualist Formula is a revised version of Kant’s Formula of Universal Law: “Act only in accordance with that maxim through which you can at the same time will that it become a universal law” (Kant 1785, AK 4:421). Parfit also considers several other formulations and interpretations of Kant’s categorical imperative, although ultimately he does not include these in the Triple Theory. Of these, the Consent Principle is particularly interesting for the purposes of this paper, since it inspires a crucial revision of the Kantian Contractualist Formula which renders the Kantian Contractualist Formula inconsistent with Parfit’s own metaethical view of reasons – or so I will argue. In addition, Parfit’s discussion of the Consent Principle includes comments on the question why moral principles are not superfluous, something that I will return to in the end of this paper.

When it comes to reasons, Parfit’s view is that there are two distinctive viewpoints – a partial and an impartial viewpoint. In some cases, as when we are choosing between spending money on an evening’s entertainment or giving the money to an effective aid agency, what we have most reason to do from a partial viewpoint differs from what we have most reason to do from an impartial viewpoint. Parfit claims that we can compare reasons presented from a partial viewpoint with reasons that are grasped from an impartial viewpoint, but only very imprecisely. Hence, when one of “two possible acts would make things go in some way that would be impartially better, but the other act would make things go better either for ourselves or for those to whom we have close ties, we often have sufficient reasons to act in either of these ways” (p. 137). This is called a *wide value-based objective view* of reasons.^1^

My objection to the account presented in *On What Matters* does not turn on whether it is right to adopt this wide value-based objective view. Nor does it concern whether it is right to revise moral principles if they entail that in some cases we have sufficient reasons to act wrongly. Instead, my objection is that you cannot endorse:

1. a moral principle which entails that agents have sufficient reasons to act wrongly in some important cases.

and simultaneously claim that:

(2) moral principles should be rejected or revised if they entail that agents have sufficient reasons to act wrongly in some important cases.

I will argue that a wide value-based objective view entails that we sometimes have sufficient reasons to act in ways that moral principles such as the Consent Principle or Kantian Contractualist Formula classify as wrong. In important cases, these principles imply that it is wrong to do what is partially best. Still, the agent has sufficient reasons to perform this act. The Consent Principle and the Kantian Contractualist Formula share a requirement that renders them inconsistent with a wide value-based objective view of reasons; they require that we consider the partial and impartial reasons of *each person*. By contrast, a wide value-based objective view only asks us to consider the partial and impartial reasons of *the agent*.

In the second section I will seek to show that Parfit does indeed hold that moral principles should be rejected or revised if they entail that agents have sufficient reasons to act wrongly in some important cases. The next section elaborates Parfit’s wide value-based objective view of reasons further. The forth and fifth sections concern the Consent Principle and the Kantian Contractualist Formula, respectively. Here I will continue the line of argument from the first section, and give some examples in which the method of revising and rejecting is employed, as well as arguing that in some important cases we might have sufficient reasons to act in ways that these formulas imply to be wrong. I will also explain why I think that the requirement that we have to consider the reasons of each person has implications that are inconsistent with a wide value-based objective view of reasons. In the final section I will make some suggestions as to how this inconsistency might be resolved. Yet it seems to me that once this inconsistency is resolved, the moral principles become substantially superfluous – in the sense that it would be possible to deduce what we ought to do without referring to those principles. Nonetheless I think that the moral principles might have another valuable feature, namely explanatory force. Moral principles can be used to illuminate what we have reasons to do.

## Revising Principles

There are at least two reasons for thinking Parfit holds that moral principles should be rejected or revised if they entail that agents have sufficient reasons to act wrongly in some important cases. First, he frequently uses this idea strategically. In this paper, I will consider a small variety of cases in which Parfit does just this. Typically, he considers cases where the moral principle in question implies that an act is wrong, but where the agent arguably has sufficient reasons to perform this act. Of course, you could regard such cases as indicating simply that Parfit takes the possession of sufficient reasons to act wrongly as a pro tanto reason (rather than a decisive one) to reject the moral principle with these implications. This brings us to the next reason.

Second, Parfit thinks that the notion of what we have sufficient reasons to do is more fundamental than the notion of what moral principles require of us. Hence, moral principles can be evaluated and rejected if they are inconsistent with what we have sufficient reasons to do. Before embarking on his treatment of the moral formulas he considers, Parfit addresses what he regards as the profoundest problem of morality: Can we have sufficient or decisive reasons to act wrongly?^2^ Consider the questions:

1. What do I have most reason to do?
2. What ought I morally to do? (p. 147).

Parfit claims that the first question is “wider, and more fundamental” (p. 147). If we often had decisive reasons to act wrongly, “that would undermine morality” (p. 147). Given this, we have a motive to revise moral principles in order to make them less conflicting with what we have reason to do. As an example Parfit considers the moral principle of act consequentialism, a principle which entails, for instance, that we should kill someone if this would be the only way to save many other people. Parfit thinks that this conclusion is counter-intuitive, and argues that there might still be sufficient (non-consequentialist) reasons for me not to kill this person. So, if act consequentialism is correct, we have sufficient reasons to act wrongly in some important cases. If we want act consequentialism to retain its normative force in such situations, we will need to revise the theory so as to make it consistent with what we have sufficient reasons to do (p. 143–4). That we ought to reject or revise moral principles when their implications are in conflict with what we have sufficient reasons to do in some significant cases can be derived straightforwardly from his view that what we have sufficient reasons to do is more fundamental than what we have reason, morally, to do.

Although this paper focuses on cases in which the agent has sufficient reasons to act wrongly, this is not the only grounds for rejecting moral principles, according to Parfit. An allegedly supreme moral principle can also be legitimately rejected if it fails to imply that an act is wrong when there are sufficient reasons for us to not perform this act. It is in this vein, for instance, that Parfit ultimately rejects the Consent Principle since “[s]ome acts are wrong even though everyone could rationally consent to them” (p. 211).^3^

## Wide Views on Reason

In order to illustrate Parfit’s wide value-based objective view of reasons we can look at one of his many examples. Suppose that:

*Case One*, I could either save myself from some injury, or act in a way that would save some stranger’s life in a distant land. (p. 137).

In this case, one action is best for me and another action is best from an impartial viewpoint. Parfit maintains that the reasons that stem from these different viewpoints can be compared, but only very imprecisely. For example, it would be impossible in Case One to decide an exact amount of injury which would make my self-interested reasons to avoid injury decisively stronger than my reasons to save the stranger’s life. I would have sufficient reasons for acting in either way “whether my injury would be as little as losing one finger, or as great as losing both legs” (p. 138). However, there will be some point at which my injury is not severe enough to give me sufficient reasons to avoid injury rather than saving the stranger’s life – for example, a small flesh wound. Parfit calls this view, or rather views of this kind:

*Wide value-based objective views:* When one of our two possible acts would make things go in some way that would be impartially better, but the other act would make things go better either for ourselves or for those to whom we have close ties, we often have sufficient reasons to act in either of these ways. (p. 137).

The word “often” is meant to capture the idea that reasons stemming from the two different viewpoints are only imprecisely comparable. Relatedly, a *wide* view of reasons indicates that the view includes both partial and impartial reasons.^4^ In addition, we can note that Parfit holds that our partial reasons are not only self-interested but also involve those to whom we have close ties. He plausibly claims that we have partial reasons to care about our future self, and that “[m]ost of us have partly similar relations to some other people, such as our close relatives, and those we love” (p. 136). Such reasons stem from the same viewpoint as self-interested reasons.

## The Consent Principle

In this section I will concentrate on two things. First, I will give a brief account of Parfit’s revisions of Kant’s Formula of Humanity, revisions that eventually result in the Consent Principle. My aim is to make it evident that Parfit’s general method is to modify moral principles if they entail that we have sufficient reasons to act wrongly. Second, I will argue that in many cases we will have sufficient reasons to act in a way that the Consent Principle describes as wrong. In these cases the Consent Principle typically implies that it is wrong not to do what is impartially best, yet there are sufficient reasons for doing another act that would make things go better either for the agent or for those to whom the agent has close ties. I will also explain why I think that the impartial implications the Consent Principle entails emerge from the requirement that we consider not only what *the agent* has sufficient reasons to do, but what *each person involved* has sufficient reasons to consent to.

### Revising the Formula of Humanity

*The Formula of Humanity:* We must treat all rational beings, or persons, never merely as a means, but always as ends.^5^ (p. 177).

Parfit considers and rejects several interpretations of this formula. One that he considers with great care originates in Kant’s claim that in order to treat people as ends we must never treat them in ways to which they could not consent. This idea can in turn be interpreted in several ways. Parfit considers O’Neill’s (1989) and Korsgaard’s (1996) interpretation that coercion and deception are the most fundamental forms of wrong-doing to others since people cannot consent to being coerced or deceived (p. 178). On Parfit’s reading of this interpretation coercion and deception are always wrong.^6^ As a counter-example Parfit asks us to consider:

*Fatal Belief:* I know that, unless I tell you some lie, you will believe truly that Brown committed some murder. Since you could not conceal that belief from Brown, he would then murder you as well. (p. 178).

Considering this and similar cases where it seems plausible that we have sufficient reasons to deceive or coerce someone, Parfit concludes that “[t]hough deception and coercion are often wrong, what makes them wrong is not, I believe, the fact that these are acts whose nature makes consent impossible” (p. 179). This is characteristic of Parfit’s method: he rejects O’Neill’s and Korsgaard’s interpretation because it has the result that too often we would have sufficient reasons to act wrongly. The line of argument is also characteristic of Parfit’s method in another way: his principal aim is not to give the most exegetically correct interpretation of Kant, but instead to arrive at an interpretation that is close to what could be considered a correct or plausible moral principle.

Having considered several different understandings of Kant’s Formula of Humanity Parfit concludes that the Consent Principle is the most plausible interpretation of this formula.

*The Consent Principle:* It is wrong to treat anyone in any way to which this person could not rationally consent. (p. 181).

This principle refers, not to actual consent, but rational consent. That is, what it is to act wrongly can be analysed in terms of what the relevant persons would rationally consent to if the relevant reason-giving facts were clear to them.

### The Implications of the Consent Principle

To understand how the Consent Principle works we can consider the example:

*Earthquake*, two people, *White* and *Grey*, are trapped in slowly collapsing wreckage. I am a rescuer, who could prevent this wreckage from either killing White or destroying Grey’s leg.^7^(p. 185).

These people are both strangers to me, and there are no morally relevant differences between them. About this precarious situation Parfit writes:

We ought, I have claimed, to accept some *wide value-based objective* theory. […] If Grey could choose how I would act, she would have sufficient reasons, I believe, to make either choice. (p. 186).

Grey can rationally consent to me saving her leg since this is what is partially best for her. She can also rationally consent to me saving White, sacrificing her own leg, since this is what is impartially best. White, by contrast, only has sufficient reasons to consent to me saving her life. From her perspective, this is the outcome that is both partially and impartially best.^8^ There is too great a difference between White’s loss and Grey’s, so White does not have sufficient partial reason to consent to me saving Grey’s leg instead, as showed in Table 1.

**Table 1:** Reasons involved in Earthquake

	…has sufficient reasons to rationally consent to me saving Grey’s leg, sacrificing White’s life	…has sufficient reasons to rationally consent to me saving White’s life, sacrificing Grey’s leg.
Grey…	Yes, from a partial viewpoint	Yes, from an impartial viewpoint
White…	No	Yes, from a partial and from an impartial viewpoint

Since the only action that everyone could rationally consent to is saving White’s life at the cost of Grey’s leg, this is the action I – the rescuer – ought to choose. This seems in line with a wide value-based objective view of reasons. We consider each person’s reasons, both partial and impartial. The relevant persons in this case are White and Grey; the partial reasons of the rescuer are negligible.

To further illustrate the Consent Principle, it seems clear that it rules out rational egoism, i.e. the thesis that “we always have most reason to do whatever would be best for ourselves” (p. 130). If we accepted rational egoism Grey would no longer have sufficient reasons to rationally consent to us saving White’s life. Since rational egoism precludes all reasons for consenting to what would be the impartially best outcome, White could only consent to me saving her life and Grey could only consent to me saving her leg. The Consent Principle then entails that whatever the rescuer does in this situation, it would be wrong. That is an unacceptable conclusion according to Parfit, and he therefore concludes that rational egoism is incompatible with the Consent Principle.^9^

The problem with the Consent Principle, as I see it, is not that it invites egoism (or partialism). Instead, it is that in many important cases this principle implies that it would be wrong for the agent to act on his or her own partial reasons, contrary to a wide value-based objective view. This problem will typically arise when (1) the agent is one of the morally relevant persons in the situation, and (2) there are other people in the situation – and the more there are, the greater inconsistency. To illustrate this we can look at Parfit’s example Self:

*Self:* I am trapped with White in slowly collapsing wreckage. I could save either White’s life or my leg. (p. 207).

This example is obviously quite similar to Earthquake. I have sufficient impartial reasons to save White’s life and sufficient partial reasons to save my own leg. White on the other hand apparently lacks sufficient reasons (partial and impartial) to agree to me saving my leg at the cost of her life. Hence the Consent Principle would imply that it is wrong of me not to save White’s life. Parfit acknowledges this conclusion, but he also suggests that one important difference between this and the Earthquake case is that White might have reasons to consent to *me saving my own leg* (p. 208). This reason did not appear in Earthquake, since the rescuer was not herself one of the trapped persons. As I interpret Parfit, this would count as an impartial reason.

Still, the difference between me losing my leg and White losing her life is perhaps too great, and this entails that White still cannot rationally consent to me saving my own leg. However, White could rationally consent to me saving my leg in a similar case where the differences are smaller. So far, I think this example captures a similar intuition to the one considered when we discussed Case One, where I could either save myself from some injury or act in a way that would save some stranger’s life in a distant land. Partial and impartial reasons can only be compared imprecisely, and therefore we should expect this kind of discussion to arise.

There is, however, one important difference. The Consent Principle involves the requirement that *the other person* must have sufficient reasons to consent to my action, a requirement that a wide value-based objective view of reasons lacks. For example, when discussing Case One the only question was what I *– the agent* – would have sufficient reasons to do. It is the requirement to consider the other persons’ reasons that ultimately leads the Consent Principle to diverge from the wide value-based objective view. This divergence is most obvious in cases where many people are involved. Another example might help illustrate this.

*Aid Agency:* I could either spend $200 on some evening’s entertainment, or give this money to some efficient aid agency, such as Oxfam, which would use this money to save some poor person in a distant land from death, blindness, or some other great harm. (p. 209).

Parfit says the Consent Principle seems to imply that we should give the money to Oxfam, since the poor person could not rationally consent to us spending the money on entertainment. We can also add that if this particular person would be saved by my choosing to give the money to the aid agency, next time I intend to spend money on evening entertainment there will very probably be another person that could be saved in a similar manner. It seems that in cases like Aid Agency we often have sufficient reasons to act in a way that the Consent Principle implies is wrong. Therefore, this principle has to be revised or rejected. Parfit does suggest some revisions in the light of this example, but he is reluctant to actually accept these revisions.

To summarise the discussion so far, the Consent Principle demands that we consider what *each person* has partial and impartial reasons to consent to, while a wide value-based objective view would only require us to consider the partial and impartial reasons of *the agent*. In many-person cases like Aid Agency, where the agent is one of the potential beneficiaries, the agent will often have sufficient partial reasons to act wrongly. This is a problem if – like Parfit – one rejects or revises moral principles implying that a person might have such reasons in significant cases like this. In cases like Self, where only one person besides the agent is involved, it can often be argued plausibly that the Consent Principle will have the same implications as a wide value-based objective view of reasons *even though* the individual facing the decision has to consider the reasons of all persons involved. These solutions are, however, deceptive in the sense that they hide the aspect of the Consent Principle that will inevitably introduce an inconsistency between what this principle implies to be wrong and what we have sufficient reasons to do on a wide value-based objective view of reasons.

Although Parfit concludes that we have “strong reasons to accept some version of the Consent Principle” (p. 211), he does not think that it can be the supreme moral principle.^10^ We can now move on and consider a moral formula which Parfit does consider to be such a principle: the Kantian Contractualist Formula. As we will see, this formula shares some problematic features with the Consent Principle, and hence cannot be coherently combined with a wide value-based objective view.

## The Formula of Universal Law

A familiar formulation of Kant’s categorical imperative is the Formula of Universal Law: “Act only in accordance with that maxim through which you can at the same time will that it become a universal law” (Kant 1785, p. AK 4:421). This is the Kantian formula that Parfit eventually incorporates into his Triple Theory, or rather a modified version of it. In this section I will first give a brief account of the revisions Parfit suggests and eventually adopts, leading to the Kantian Contractualist Formula. As in section 3, the purpose of presenting these revisions is twofold. I present them partly to give an idea of the implications of the formula, and partly because I want to make the case that Parfit’s preferred philosophical method for approaching moral principles is to revise them if they entail that we have sufficient reasons to act wrongly. I will also argue that the Kantian Contractualist formula shares some important features with the Consent Principle.

### Revising the Formula of Universal Law

One interpretation of the Formula of Universal Law is:

*the Moral Belief Formula:* It is wrong for us to act on some maxim unless we could rationally will it to be true that everyone believes that such acts are morally permitted.^11^ (p. 286).

Parfit thinks that the impartiality in the Moral Belief Formula is too weak. Consider the following case:

*Unjust Punishment:* Unless *White* goes to the police and confesses, *Black* will be convicted and punished for some crime that White committed. Though White knows this fact, he does nothing. (p. 330).

The problem here is that White could rationally will everyone to act on the maxim ‘let others be punished for my crimes’, since this would entail that he can avoid many years in prison. Moreover, since this is a rare situation, it is unlikely that he will ever be in a situation where he himself would be wrongly imprisoned. Hence the Moral Belief Formula will incorrectly imply that it is wrong for White to go to the police and confess his crime. This is called the Rarity Objection. According to Parfit, another case in which the Moral Belief Formula delivers the wrong result is:

*Murderous Theft:* While travelling across some desert, *Grey* and *Blue* have both been bitten by some snake. Blue has prudently brought some drug that is an antidote to this snake’s lethal poison. Grey cannot save his life except by stealing Blue’s drug, with the foreseen result that Blue dies. (p. 331).

In Murderous Theft, the problem is that it is better for Grey to act on the maxim ‘do whatever would be best for me’. Grey could rationally want everyone to act on this maxim, since he would be better off in a world where everyone did so than he would be if he refrained from stealing the drug. This is called the High Stakes Objection.^12^

Reflecting on these cases (and some others), Parfit concludes that the Consent Principle has more attractive implications than the Moral Belief Formula. Since the Consent Principle demands that we consider each person’s reasons, it does not incorrectly imply that it would be wrong for White to go to the police and confess his crime or that it would be wrong for Grey not to steal Blue’s drug. The reason why the Moral Belief Formula has incorrect implications is – according to Parfit – that, when we apply it, we study the dilemma only from the agent’s point of view. The remedy is to take the other person’s perspective into account. This way, we cannot disregard what Black and Blue have reasons to rationally will in the circumstances.

*The Revised Moral Belief Formula:* It is wrong to act in some way unless everyone could rationally will it to be true that everyone believes that such acts are morally permitted.^13^ (p. 340).

This fits well with the thought that we cannot distinguish right from wrong by considering only the agent’s reasons for doing something. We have to consider what *everyone* could rationally will, both partially and impartially. The problem is that this revision invites back the problems we saw to be connected with the Consent Principle. For instance, when we apply the Revised Moral Belief Formula to many-person cases like Aid Agency we have to consider the reasons everyone would have – both partial and impartial – if they knew the morally relevant facts. This would entail that it is wrong for me to spend money on an evening’s entertainment since the poor person who could be saved from some great harm does not have sufficient reasons to rationally will that I spend the money on myself.^14^ Unfortunately, Parfit does not consider cases like Aid Agency and Self in relation to the Kantian Contractualist Formula.

Another revision Parfit (pp. 289–300) advocates – a revision included in the Revised Moral Belief Formula – is that we should abandon Kant’s notion of a maxim. He argues that there are many “maxims or policies on which it would be sometimes but not always wrong to act” (p. 295). For example, suppose that an egoist saves a child from drowning only in order to get some reward. Kant’s formulas would wrongly condemn this act, since the egoist acts on the maxim ‘do whatever would be best for me’. According to Kant’s formulas this egoist would also act wrongly when he pays his debts, takes some medicine or puts on warm clothing. Parfit argues in a familiar fashion that “Kant could not have rationally willed it to be true that no one ever tells a lie, not even to a would-be murderer who asks where his intended victim is” (p. 292). He considers several other cases as well, but his point is that most maxims are “*morally mixed* in the sense that, if we always acted on these maxims, some of our acts would be wrong, but other acts would be permissible or even morally required” (p. 293). He concludes that Kant’s formulas should be revised in a way that does not refer to the agent’s maxim, but rather refers to the morally relevant facts. In other words: we act wrongly unless everyone could rationally will it to be true that everyone believes that such acts are morally permitted (in circumstances involving the same array of morally relevant facts).^15^ Finally, Parfit reformulates the Revised Moral Belief Formula to:

*the Kantian Contractualist Formula:* Everyone ought to follow the principles whose universal acceptance everyone could rationally will.(p. 342).

This formula clearly requires us to consider what everyone could rationally will rather than what the agent can rationally will. It is less obvious that it includes the requirement that we consider the morally relevant facts (in contrast with maxims). However, Parfit explains, “When people believe that some kind of act is morally permitted, they accept some principle that permits such acts” (p. 341). Further, the kind of act at issue is defined by the circumstances in which the act is performed and the morally relevant facts these circumstances involve. Parfit ends his discussion of the Formula of Universal Law by saying that the Kantian Contractualist Formula “might be what Kant was trying to find: the supreme principle of morality” (p. 342).

### Taking Stock

The Kantian Contractualist Formula and the Consent Principle share some important requirements. They require:

- that we consider the morally relevant facts involved in the particular situation where the act is performed,
- that we consider what *everyone* has sufficient reasons to rationally will or consent to, and.
- that we consider what everyone has both *sufficient partial and sufficient impartial* reason to rationally will or consent to.

A wide value-based objective view of reasons does not generate the second of these requirements.^16^ It requires us to consider only the partial reasons the agent has, together with any impartial reasons involved in the situation. This difference entails that the implications of the Kantian Contractualist Formula and the Consent Principle differ from those of a wide value-based view, at least in many-person cases where the agent is one of the morally relevant persons. This is what I am referring to when I claim that the Consent Principle and the Kantian Contractualist Formula are inconsistent with a wide value-based objective view of reasons. Put differently, my claim is that these principles entail that agents have sufficient reasons to act wrongly in some important cases.

In addition, it is evident that Parfit revises or rejects moral principles if they entail that we have sufficient reasons to act wrongly in some important cases. This response is an upshot of his view that reasons are more fundamental than moral principles, and of his intention to arrive at moral principles which matter in the sense that they do not imply too often that we have sufficient reasons to act wrongly. My objection is that you cannot adhere to (1) a moral principle entailing that agents have sufficient reasons to act wrongly in some important cases while, at the same time, holding that (2) moral principles should be rejected or revised if they entail that agents have sufficient reasons to act wrongly in some important cases.

## Solutions

In this final section I will discuss some possible ways to address the objection summarized in the end of the previous section. First, I will discuss whether it might after all be conceivable that we sometimes have sufficient reasons to act wrongly. This seems to be Parfit’s preferred solution. To jump ahead, I conclude that this is conceivable when we are dealing with the principles which need to be revised or rejected (act-utilitarianism is such a principle on Parfit’s view), but that it is not an option when we consider supreme moral principles such as those included in the Triple Theory. Supreme moral principles are supposed to be fully revised in such a way that they cease to entail that we have sufficient reasons to act wrongly.

Second, I suggest another solution. We ought to resist the revision of the Kantian Contractualist Formula introducing the claim that we have to consider the reasons of everyone rather than simply the reasons of the agent. If we resist this revision, the Kantian Contractualist Formula will not entail the inconsistencies with a wide value-based objective view I have considered in this paper. However, if we resist this revision at least two other problems surface. First, we cannot deal with cases like Unjust Punishment and Murderous Theft in the way Parfit does. Here, I will argue that we will be in a position to solve such cases if we accept Parfit’s other revision: that we should abandon the notion of a maxim and instead consider the morally relevant facts. Second, the Kantian Contractualist Formula will become substantially superfluous. It will not add anything normatively substantial that is not already implied by a wide value-based objective view of reasons. There is, however, a solution to this problem as well, and this is to insist that the moral principles are not superfluous because they are explanatory. They have a heuristic value for us when we are trying to figure out what morality requires.

Finally, I will briefly discuss some other possible solutions. One option would be to revise the wide value-based objective view on reason in order to render it consistent with the Kantian Contractualist Formula. Another option would be to argue that when a moral principle entails that agents might reasons to act wrongly, this is not necessarily tantamount to saying that we have decisive reasons to revise or reject the moral principle. It could suffice to say that when this is the case, we have a pro tanto reason to revise or reject the moral principle.

### Accepting Sufficient Reasons to Act wrongly

One solution to the inconsistency problem at hand would be to deny that the inconsistency is a problem. According to this solution, we could accept that you might have sufficient reasons to act wrongly. In a mail correspondence between Parfit and Wlodek Rabinowicz some years ago,^17^ Parfit clarifies that he thinks that you might have such reasons, and thus that there is no inconsistency between the moral principles and a wide value-based objective view. This solution is also indicated on several occasions in *On What Matters*. For instance, Parfit argues that if what is wrong is decided by act-utilitarianism, it would be wrong for us not to sacrifice our life in order to save the lives of several strangers. However, he continues, we might have sufficient reasons to act wrongly in cases like this (p. 148). Similarly, Parfit argues that we might have sufficient reasons to act in a way that a moral formula such as act consequentialism classifies as wrong if this “wrong act was our only way to save from great pain or death, not ourselves, but our close relatives, or other people whom we love” (p. 143). As a further example he argues that:

We might also reject Sidgwick’s claim that we could always rationally do whatever we knew would make things go best. As an *Act Consequentialist*, Sidgwick believes that such acts would always be morally right. Most of us reject this view, since we believe that certain acts would be wrong even if they would make things go best. The wrongness of such acts, we might claim, would often give us decisive reasons not to act in these ways. (p. 136).

In arguments like this there are two types of wrong involved: what is wrong according to act consequentialism and what reasons imply to be wrong. This slightly obscures the line of argument.^18^ Parfit’s point is that if what is wrong is decided by a moral principle such as act consequentialism, we might sometimes have sufficient and even decisive reasons to act wrongly.

In the end, act consequentialism is not one of the principles incorporated in the Triple Theory. Instead this principle is rejected since it undermines morality. It “conflicts too often, or too strongly, with our intuitive beliefs about which acts are wrong” (p. 417). The Kantian Contractualist Formula differs from act consequentialism in this respect, since, as Parfit sees it, it is one of the supreme formulas that conflict neither too often nor too strongly with what we have sufficient reasons to do. And the Consent Principle is close to being such a principle.

It is hardly surprising that a principle which, at the end of the day, we find suitable to reject or revise might entail that we sometimes have sufficient reasons to act wrongly. We can consider an analogous case in the natural science. A Newtonian understanding of physics implies that sunbeams always run in a straight line. However, at the beginning of the twentieth century, carefully conducted observations showed that sunbeams in fact bend through space under the influence of intense gravity. (This observation is famous for settling the issue of which of Einstein’s relativity theory or Newton’s mechanistic conception of physics is the more correct.) Under such circumstances, we should conclude that there is something incorrect about our scientific principle, not with our observation. The same can be said about candidate supreme moral principles.

Parfit does not consider whether the Kantian Contractualist Formula entails that we sometimes have sufficient reasons to act wrongly. Presumably, this formula is supposed not to allow for such acts. Instead he addresses the question when he considers the Consent Principle in relation to the extreme demands of morality in cases like Self and Aid Agency. Self*,* Parfit argues, is a case where we might have sufficient reasons to act wrongly. If the stakes were too high (as they may be in this situation) White would not have sufficient reason to rationally consent to me saving my own leg. In that case it would be wrong not to save White’s life, according to the Consent Principle. Still, I might have sufficient partial reason to save my own leg. Consequently, the Consent Principle might be in conflict with what I have sufficient reason to do in cases like Self*.* A similar point can be made about Aid Agency. Interestingly, Parfit argues that the Consent Principle “may be too demanding, and there may be some other ways in which it should be revised” (p. 211). Yet he does not revise the Consent Principle in the light of these examples.

If we consider what the Kantian Contractualist Formula entails in cases of extreme demands, we seem to get the same results. Since this formula requires that we consider what *everyone* could rationally will, we have to consider what White could rationally will in Self. As before, if the stakes were too high, White could not rationally will that I save my own leg rather than White’s life. White lacks sufficient reasons to do so both from a partial and an impartial viewpoint. Yet, I might have sufficient reasons to save my own leg from my own partial viewpoint. Hence, I have sufficient reasons to act wrongly, and it seems like also the Kantian Contractualist Formula is too demanding.

To conclude, when it comes to our supreme moral principles we cannot be satisfied if they entail that we may have sufficient reasons to act wrongly. For this reason, act consequentialism cannot be a supreme moral principle. Neither can the Consent Principle, nor the Kantian Contractualist Formula.

### Avoiding Sufficient Reasons to Act wrongly

A version of the Kantian Contractualist Formula that is not inconsistent with a wide value-based objective view of reasons in the sense I have been discussing is perfectly conceivable. Such a version would be identical with Parfit’s Kantian Contractualist Formula except for one thing: it would lack the requirement to consider what *everyone* could rationally will. Instead it would require merely that we consider what *the agent* could rationally will. In his line of argument, Parfit proposes and rejects several versions of the Moral Belief Formula (on which the Kantian Contractualist Formula is modelled). One of these versions is especially interesting, since it lacks the requirement that we have to consider what everyone could rationally will but includes the other revisions Parfit advocates.

*The Moral Belief Formula 2*: We act wrongly unless we [ourselves] could rationally will it to be true that everyone believes such acts to be morally permitted.^19^(p. 296).

This formula is consistent with a wide value-based objective view of reasons and so avoids being too demanding in situations like Aid Agency and Self. A potential problem is that we could lose the attractive implications of the Kantian Contractualist Formula in cases like Murderous Theft and Unjust Punishment. Is it possible to secure these attractive implications in some other way?

When it comes to Murderous Theft – where Grey only can save his life by stealing a drug from Blue with the foreseen consequence that Blue dies – Parfit argues that the original Moral Belief Formula mistakenly implies that Grey is permitted to steal the drug. In this case, Grey could act on the maxim ‘Do whatever would be best for me’, since rationally he could will to live in a world where everybody acts on this maxim rather than to be dead. We could instead consider what outcome emerges if we apply the Moral Belief Formula 2. In the case of Murderous Theft, we would no longer need to consider what kind of society Grey could rationally will that he lives in when he comes home from the desert. For he would not be acting on the general maxim ‘Do whatever would be best for me under any circumstances’. Rather he would be acting on something more specific, namely what he could rationally will that everyone believes under these specific circumstances. Perhaps we could accept that it is permitted to steal an antidote under such extreme circumstances.

If we do not like this conclusion, this is probably because we think that the impartial reasons not to steal the antidote are sufficiently stronger than Grey’s partial reasons to steal it. Compare this case to Case One, presented in the beginning of the paper. In that case I could either avoid an injury or act in a way that would save a stranger’s life in a distant land. Parfit argued that a wide value-based objective view entails that there will be some point at which my injury is simply not severe enough to give me sufficient reasons to avoid it. For instance, a small flesh wound would not give me sufficient reasons to avoid that injury instead of saving the stranger’s life. Likewise, Murderous Theft could be one of those rare cases where one of two possible acts would be impartially better and the other act would be better for the agent, but where the agent does *not* have sufficient reasons to act in either of these ways. Grey’s partial reasons to steal the drug is outweighed by the impartial reasons not to steal the drug. Hence, Grey cannot rationally will that it is true that everyone believes such acts to be morally permitted, and the Moral Belief Formula 2 would entail that he acts wrongly if he steals the drug.^20^ There is no need to involve Blue’s partial reasons as the Kantian Contractualist Formula does in order to argue that it is wrong for Grey to steal the antidote.

I think that we could argue along similar lines when it comes to Unjust Punishment. Even though the Moral Belief Formula 2 does not require White to consider Black’s partial reasons, White’s partial reasons not to confess to the police could, in the circumstances, be outweighed by some impartial reasons. One such reason may be, for instance, that one should not knowingly allow a person to be punished for a crime he did not commit if one can prevent this from happening. This reason is probably especially important when one has committed the crime oneself. In addition, there may be sufficient partial reasons for White to confess. For instance, it could be that White values Black’s friendship to the extent that he has sufficient partial reasons to confess to the crime.^21^

The general strategy to solve cases like Unjust Punishment or Murderous Theft would be to argue either 1) that in such rare and extreme cases there are in fact sufficient reasons to do what first appeared to be wrong, or 2) that there are impartial reasons, or hitherto neglected partial reasons from the agent’s point of view, which outweigh the apparently sufficient partial reasons to act in the way we consider wrong. It will be more complicated to see what morality requires of us in many such cases if we abandon the requirement to consider the reasons of each involved person. However, if the case we are trying to solve is morally complicated, we should not be surprised if a satisfying answer requires extensive analysis.

One last worry arises when the Kantian Contractualist Formula is amended to be consistent with a wide value-based objective view of reasons. The formula becomes substantially superfluous in the sense that it would be possible to deduce what we ought to do without referring to those principles. A possible objection would be that even if the formula is true, “we do not need this principle as a criterion, nor is this principle explanatory” (p. 190). Parfit answers such an objection in connection with the Consent Principle, arguing in this case that the principle is not superfluous since it has substantial implications. The Consent Principle “would have most importance when we must choose between many possible acts that would have significant effects on many people, whose interests or aims conflict” (p. 191). Aid Agency is one such case, and here the implications of the Consent Principle will differ from what a wide value-based objective view of reasons implies. As I have argued, these substantial implications emerge as a consequence of the requirement that we have to consider what *each person* or *everyone* could rationally consent to. This requirement generates substantial implications simultaneously, as it makes the formula inconsistent with a wide value-based objective view of reasons. When we have revised our moral formulas (or our view on reasons) so that they are consistent, we cannot argue that our formulas add something substantial. They are substantially superfluous. The moment they add something substantial beyond what our view on reasons already entails, they will become inconsistent with our view of reasons and should therefore be revised or rejected. We can, however, argue that there is another important way in which the formulas are valuable. They have an explanatory value that is vital for our moral understanding. They might, for example, facilitate our reasoning in morally complicated cases, or aid our development of moral understanding.

### Other Possible Solutions

My aim here is not to show that the Moral Belief Formula 2 is the supreme moral principle, or that the solution I proposed under the headline “Avoiding sufficient reasons to act wrongly” is the only possible one. It is to show that there is at least one plausible way to develop the Kantian Contractualist Formula along lines that are consistent with a wide value-based objective view of reasons. If it is judged to be desirable to keep the requirement that we must consider the reasons of everyone (and it may, for example, be useful in cases like Earthquake to consider the reasons of each person), there is another solution. The Kantian Contractualist Formula can be retained as it is, and instead the wide value-based objective view of reasons can be amended so that it now involves the idea that we have to consider the reasons of each person involved. Whichever solution is ultimately adopted, it must secure the outcome that the position taken on reasons is consistent with the preferred moral principle.

What about the Consent Principle? Can it be revised in a way that does not entail that we may have sufficient reasons to act wrongly? Unfortunately, the Consent Principle seems to be beyond repair in this respect. The idea that what other persons could rationally consent to must be considered fundamental for this principle. To erode or drop this idea would be tantamount to giving up the principle. It seems, then, that the best way to render this principle consistent with a wide value-based objective view is to revise the wide value-based objective view.

There is yet another solution. The objection I put forward in this paper is that you cannot adhere to (1) a moral principle entailing that agents have sufficient reasons to act wrongly in some important cases while, at the same time, holding that (2) moral principles should be rejected or revised if they entail that agents have sufficient reasons to act wrongly in some important cases. You could, however, revise (2) and claim (2*) that *there is a* pro tanto *reason* to reject or revise moral principles if they entail that agents have sufficient reasons to act wrongly in some important cases. This would open up for introducing a compelling argument that shows that the Kantian Contractualist Formula should be accepted, all things considered, even though it entails that agents have sufficient reasons to act wrongly in some important cases. Perhaps all Parfit needs is such a compelling argument. Still, if Parfit would accept this strategy, there is more work to be done. He has to show that none of the moral principles that he currently rejects on the grounds that they entail that agents might have sufficient reasons to act wrongly could be accepted after all, when everything is considered.

## Footnotes

1 Parfit uses the indefinite article in ‘a wide value-based objective view’ of reasons since he thinks that this view can be further specified in a number of ways, and he wants to include all these ways in his reasoning. He writes, “Different wide value-based objective views make conflicting further claims about when it would not be true that we had sufficient reasons to act in either of these ways. We ought, I believe, to accept some view of this kind.” (p. 137).

2 This question, as well as Parfit’s answers to it, is inspired by Henry Sidgwick’s analysis in *The Methods of Ethics* (1874). Sidgwick thought that (here in Parfit’s phrasing) “We always have most reason to do whatever would be impartially best, unless some other act would be best for ourselves. In such cases, we would have sufficient reasons to act in either way. If we knew the relevant facts, either act would be rational” (p. 131). This is called the dualism of practical reason. The profoundest problem arises when we have to choose between two acts, of which one is impartially best but the other would be better for ourselves. In such cases our duty conflicts with our self-interest, to use Sidgwick’s phrasing. The problem is – at best – that in such cases morality will give no guidance as how to act. At worst, the problem is that we cannot avoid acting irrationally in such cases. Whichever act we choose, it will be irrational either from a self-interested standpoint or from the point of view of the universe (again, to use Sidgwick’s words). As a historical background, Sidgwick lost his faith in God while writing *Methods of Ethics* and hence lost the at the time most common argument for how morality and egoism is combined; the argument that if you don’t act morally you will be condemned to hell in the afterlife. His pessimism about ever resolving the profoundest problem is best expressed in the concluding sentence of the first edition of *the Methods of Ethics*: “But the Cosmos of Duty is thus really reduced to a Chaos: and the prolonged effort of the human intellect to frame a perfect ideal of rational conduct is seen to have been fore-doomed to inevitable failure” (Sidgwick 1874, p. 473). Sidgwick once noted that *The Methods of Ethics* begins with the word “ethics” and ends with the word “failure” (Hayward 1901, p. 187). This was naturally intended as a joke, but as in many good jokes there was something more substantial to it as well.

3 It seems question-begging to state that acts can be wrong even though everyone could rationally consent to them. The Consent Principle dictates what acts we denote as ‘wrong’, at least to the extent that we accept it. I think Parfit expresses his argument with insufficient care. Probably, what he meant was that the Consent Principle fails to identify some acts as wrong, even though there are sufficient reasons for the agent to refrain from performing these acts. I come back to this in footnote 10.

4 For the sake of the discussion, I will assume that reasons are value-based and objective.

5 As Kant puts it (here translated by Wood (2002)): “Now I say that the human being, and in general every rational being, *exists* as end in itself, *not merely as means* to the discretionary use of this or that will, but in all its actions, those directed toward itself as well as those directed toward other rational beings, it must always *at the same time* be considered as an *end*” (Kant 1785, p. AK 4:428). Kant also writes: “Act so that you use humanity, as much in your own person as in the person of every other, always at the same time as end and never merely as means” (Kant 1785, p. AK 4:429).

6 Parfit’s reading of Korsgaard is not particularly charitable. In her paper “The Right to Lie: Kant on Dealing with Evil” (1986) Korsgaard argues that there are situations in which lying is the right thing to do, all things considered.

7 Parfit considers another example called *Lifeboat* (p. 186) with a similar structure. Both of these examples can also be found in “Justifiability to Each Person” (Parfit 2003, pp. 369, 376).

8 We assume that Grey and White are not close or linked by family ties.

9 I agree that the Consent Principle is inconsistent with rational egoism. However, it seems to me that if rational egoism were true, I, as a rescuer, would not be concerned by the precarious situation of White and Grey in the first place. I would act on my egoistic (or partial) reasons and go and buy myself a chocolate and vanilla ice cream instead (provided that this would not make me feel bad afterwards).

10 This is not because he thinks the principle would be too demanding, entailing that we too often have sufficient reason to act wrongly. Instead it is because “some acts are wrong even though everyone could rationally consent to them” (p. 211). According to Parfit (p. 200), one example could be voluntary euthanasia if we assume that all relevant persons could rationally consent to me carrying out the mercy killing. Examples of wrongdoing that are not ruled out by the Consent Principle, according to Parfit, also include cruelty to animals and possibly also suicide. However, I fail to see why the Consent Principle would necessarily rule out cruelty to animals, and why the Kantian Contractualist Formula would not do so. First, the Consent Principle is based on hypothetical consent, not actual consent. Surely it makes sense to say that, for instance, a dog *would* consent to being vaccinated if she knew (and understood) the relevant fact that this treatment prevents her from becoming ill, despite the fact that she *actually* struggles against her owner’s hold on her when the vet arrives. And surely it makes sense to say that she would not consent to cruel treatment if she knew more than she does and were more capable of rational thought. (I am in debt to an anonymous reviewer for stressing this point.) Second, it is true that the Consent Principle as it is formulated only applies to persons. Yet we could readily substitute, say, ‘sentient being’ for ‘person’ to avoid this problem. Moreover, I do not see why ‘everyone’ in the Kantian Contractualist Formula necessarily includes animals. It seems that this Formula might encounter the same problem as the Consent Principle in this respect. Indeed in my view none of the arguments Parfit presents for the differentiation of the Consent Principle and the Kantian Contractualist Formula succeeds. Both formulas fundamentally involve the notion that the agent has to consider the partial and impartial reasons that each person (or everyone) under the circumstances has vis-à-vis a certain act. Whether these reasons count in favour of rationally consenting to the act, or in favour of rationally willing universal acceptance of the same act, seems less important. Limits of space here mean that this issue must be taken up in a separate paper.

11 Parfit also considers numerous other moral principles which I have not discussed here, including the Law of Nature Formula, the Permissibility Formula, the Impartial Observer Formula and different versions of the Golden Rule. However, all of these formulas are eventually rejected (except the Law of Nature Formula which is very similar to the Moral Belief Formula).

12 When he considers these examples Parfit is referring, not to the Moral Belief Formula, but to the Law of Nature Formula. The latter is revised in the light of the examples, and Parfit eventually applies the same revisions to the Moral Belief Formula.

13 Scanlon (1998, pp. 170-171) earlier proposed a similar interpretation of Kant’s Formula of Universal Law.

14 The notion of ‘everyone’ is ambiguous, and the Revised Moral Belief Formula can be interpreted in at least two ways.

- *Interpretation A:* It is wrong to act in some way unless *each person involved in the situation* could rationally will it to be true that everyone believes that such acts are morally permitted.
- *Interpretation B:* It is wrong to act in some way unless *everyone currently alive* could rationally will it to be true that everyone believes that such acts are morally permitted.

Parfit says very little about how to interpret ‘everyone’ in this context. If interpretation A is correct, the Revised Moral Belief Formula will be identical with the Consent Principle in respect of those whose reasons we are to consider. In this case, the implications of Revised Moral Formula will be inconsistent with the implications of a wide value-based objective in the same way as the Consent Principle. If interpretation B is correct, it might seem that the Kantian Contractualist Formula will be even more seriously inconsistent with a wide value-based objective view than the Consent Principle is. We have to consider the reasons of the agent, of the other persons involved in the situation *and everyone else.* However, the inclusion of people who are not affected at all by the action will probably not make an otherwise permissible action wrong. Arguably, those persons will lack partial reasons to rationally will the act in question to be permitted. They will only consider the situation from an impartial viewpoint, and this viewpoint is already being considered by the agent and those affected by the action (if they know the relevant fact). Therefore, it seems that Interpretation A and Interpretation B have the same moral implications, even though they differ in content.

15 The observation that Kant’s notion of a maxim poses interpretative challenges is nothing new. Parfit refers, for example, to Wood (2002), Herman (1993), O’Neill (1975) or Hill (2002).

16 I assume that a wide value-based view involves both the first and the third requirement, or something close to them.

17 Rabinowicz was at the moment supervising my master thesis, which is where I first discussed the inconsistency between a wide value-based objective view and the Kantian Contractualist Formula. In a mail to Parfit, Rabinowicz posed some of the questions I raised in this thesis. In answering those questions, Parfit clarified that he thinks that we should accept that you might have sufficient reasons to act wrongly, and he also called attention to some of the quotations from *On What Matters* I have included in this section. One other example of when Parfit accepts that you might have sufficient reason to act wrongly is when he discusses the concept of reasons. “Though most kinds of reason are decisive only in certain cases, there may be some kinds of reason that are always decisive. On some views, for example, we always have decisive reasons not to act wrongly” (p. 32). The wording is careful and indicates that Parfit is sceptical about the idea that we always have decisive reasons not to act wrongly. It should be noted that this correspondence took place before *On What Matters* was published in 2011, and it concerned the preceding manuscript *Climbing the Mountain*. Parfit might have revised his view on this matter since then.

18 I raised a similar problem in note 3.

19 Accordingly, the Kantian Contractualist Formula would become: Everyone ought to follow the principles whose universal acceptance we ourselves could rationally will.

20 There is a fairly thick conception of ‘rationality’ that is doing a lot of work both in the Kantian Contractualist Formula and in the Moral Belief Formula 2.

21 White could also have a further reason to confess. If he does not confess, he runs the risk that he, or some of his friends, will revenge this wrong in a very unpleasant way. However, such reasons run into the wrong kinds of reasons problem. (Thanks to the anonymous reviewer for pointing this out.)
